# Visibility graph analysis for brain: scoping review

**DOI:** 10.3389/fnins.2023.1268485

**Published:** 2023-09-29

**Authors:** Sadegh Sulaimany, Zhino Safahi

**Affiliations:** Social and Biological Network Analysis Laboratory (SBNA), Department of Computer Engineering, University of Kurdistan, Sanandaj, Iran

**Keywords:** visibility graph, EEG, brain disorders, graph analysis, diagnosis

## Abstract

In the past two decades, network-based analysis has garnered considerable attention for analyzing time series data across various fields. Time series data can be transformed into graphs or networks using different methods, with the visibility graph (VG) being a widely utilized approach. The VG holds extensive applications in comprehending, identifying, and predicting specific characteristics of time series data. Its practicality extends to domains such as medicine, economics, meteorology, tourism, and others. This research presents a scoping review of scholarly articles published in reputable English-language journals and conferences, focusing on VG-based analysis methods related to brain disorders. The aim is to provide a foundation for further and future research endeavors, beginning with an introduction to the VG and its various types. To achieve this, a systematic search and refinement of relevant articles were conducted in two prominent scientific databases: Google Scholar and Scopus. A total of 51 eligible articles were selected for a comprehensive analysis of the topic. These articles categorized based on publication year, type of VG used, rationale for utilization, machine learning algorithms employed, frequently occurring keywords, top authors and universities, evaluation metrics, applied network properties, and brain disorders examined, such as Epilepsy, Alzheimer’s disease, Autism, Alcoholism, Sleep disorders, Fatigue, Depression, and other related conditions. Moreover, there are recommendations for future advancements in research, which involve utilizing cutting-edge techniques like graph machine learning and deep learning. Additionally, the exploration of understudied medical conditions such as attention deficit hyperactivity disorder and Parkinson’s disease is also suggested.

## 1. Introduction

The human brain is undoubtedly one of the most complex and mysterious organs in the human body. Understanding the neural mechanisms of brain activities has posed a significant challenge for scientists (Sporns and Honey, 2006). Specifically, the brain is a network of numerous different regions, each with its own specific function and task, constantly sharing information with each other (van den Heuvel and Hulshoff Pol, 2010). To comprehend brain function or pairwise interactions between different regions of the brain, researchers often rely on non-invasive techniques such as functional magnetic resonance imaging (fMRI), which analyses structural and functional modifications in brain disorders (Matthews and Jezzard, 2004). Additionally, researchers also make use of electroencephalography (EEG) and magnetoencephalography (MEG), which are non-invasive methods used for recording brain electrical activity, which allow them to record and analyze brain activity without harming the subject.

The utilization of time-series data obtained from non-invasive techniques such as fMRI, EEG, and MEG is one of the most valuable resources for carrying out computations and studies related to the brain (Yu et al., 2020). Several methods exist for analyzing time-series data, with one of the latest and significant ones being visibility graphs (VGs) that was proposed by Lacasa et al. (2008). This method involves mapping the time-series data to a graph, followed by performing computations on this graph. It has emerged as an important tool for gaining insights into brain function and inter-regional interactions.

The use of VGs in brain research is just one example of the many computational methods that are being developed to understand the brain. Machine learning techniques, graph theory and network methods (van den Heuvel and Hulshoff Pol, 2010), dimensionality reduction techniques, fuzzy models (Yu et al., 2020), etc. are all being used to analyze brain data and gain insights into brain function. By analyzing time-series data of brain activity, researchers are able to obtain valuable information for the diagnosis, prediction, and analysis of brain diseases or related applications. For instance, early diagnosis of a disease can slows down its progression and even lead to its cure. Nevertheless, the application of network theory to time series data is not directly possible because of non-relative structure of the data. Consequently, researchers have devised methods like VG analysis to harness the potential of network theory in analyzing and predicting brain time series data. This approach enables the exploration of new techniques and interpretations, thereby expanding the scope of research in this field.

Due to the novelty and youth of the field of VG analysis in brain researches we has chosen the scoping review to glance the topic. A scoping review is a type of literature review that aims to map the existing literature on a particular topic, identify gaps in the research, and provide an overview of the available evidence. Unlike a systematic review, which focuses on answering a specific research question using a predefined set of criteria, a scoping review is more exploratory in nature and can be used to identify research gaps and inform the development of future research questions. Scoping reviews can be particularly useful in fields where the literature is rapidly evolving or where there is a large volume of research on a particular topic (Munn et al., 2018).

In this article, we aim to provide an overview of research related to the brain’s VG, which has demonstrated significant potential for analysis and promising results. The focus of our review will be to explore the scope and breadth of brain-related research that utilizes VG analysis, with the goal of providing valuable insights and knowledge that can guide and inspire future research opportunities. The potential of the brain’s VG for analysis and research has been demonstrated by various studies. Understanding the brain and its mechanisms is crucial for advancing medical treatments, developing new therapies for neurological disorders, and improving overall human health. Our review aims to highlight the importance of VG analysis in this pursuit and its potential to revolutionize the field of brain research. Specifically, our research aims to address the following questions:

1. What is the VG, and how is it used in brain research?
2. What are the current applications of VG analysis in brain research, and what are the limitations and challenges associated with these applications?
3. What are the future research opportunities and directions in brain research utilizing VG analysis, and how can these opportunities be pursued to advance our understanding of brain function and neurological disorders?

Our paper is structured into five sections. After introduction in the first section, part 2 introduces the concept of VGs and their types, followed by an explanation of VG analysis, its definition, and purpose. Section “3. Methodology” details the research methodology, including eligibility criteria for paper selection, information sources, and the PRISMA flow diagram. In part 4, we present our findings and interpretations using various diagram formats and perspectives to enhance our analysis and deepen the understanding of the brain’s VG for disorders. Lastly, part 5 summarizes our findings, discusses future research potential, and proposes avenues for further investigation to advance knowledge in this field.

## 2. Background

### 2.1. Visibility graph

The VG is a potent tool for analyzing time series data by mapping it onto a network. In its creation method, each time series sample is represented as a node in a graph, with edges between nodes defined based on their mutual visibility. Specifically, to create an edge between two nodes (*t*_*i*_, *y*_*i*_) and (*t*_*j*_, *y*_*j*_), it must be determined whether the two corresponding time series samples can “see” each other, which is achieved through the use of an intermediate node. Here, *t_i_* stands for *i*-th point in the timeseries, and *y_i_* is the value associated with the point. This is known as visibility, and vertices are connected to each other provided that the following condition is satisfied:


(1) $$y_{n}<y_{j}+\left(y_{i},y_{j}\right)\,\frac{t_{i}-t_{n}}{t_{j}-t_{i}}.$$

**FIGURE 1:**
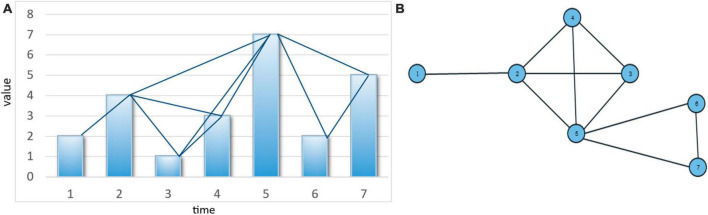
**(A)** A time series bar chart and its extracted graph (graph nodes include time points on the horizontal axis, and graph edges consist of lines connecting values that meet the condition in Equation 1, and **(B)** the resulting graph from mapping the time series.

This algorithm has wide applicability across many domains, including medicine, economics, and social sciences. By leveraging time series data in this way, it becomes possible to search for various patterns and relationships within the data, which can lead to new insights and discoveries. The resulting graph from the algorithm is a simple graph that satisfies three conditions (Figure 1): connectivity, without direction, and preserving the information of the time series data after being transformed into a graph due to the fulfillment of the condition in Equation 1. In summary, the VG approach provides a highly effective framework for analyzing time series data. Its ability to identify complex patterns and relationships within the data makes it a valuable tool for researchers and analysts in a range of fields.

The previous description pertained to the natural VG, commonly referred to as the VG. Besides, several types of VGs have been developed, each with its own specific characteristics and applications. Here, we list the most commonly used types of VGs in brain-related studies, along with a brief description of each one. We also provide a table, Table 3, that summarizes the brain disorders that have been associated with each type of VG collecting the related references:

• *Horizontal visibility graph (HVG):* similar to natural VG, but the nodes are connected by edges if the data points are visible to each other in a horizontal line of sight, offering a simplified visual representation of the time series (Luque et al., 2009).
• *Limited penetration visibility graph (LPVG):* a modified form of the VG where edges are allowed between nodes within a certain distance threshold, offering a more localized representation of the underlying patterns in a time series to reduce the effect of noise in the data (Ting-Ting et al., 2012).
• *Visibility graph similarity (VGS):* the average similarity estimate between two graphs is created with the help of mutual correlation between a sequence of degrees obtained from the time series interval in the VG (Ahmadlou and Adeli, 2012).
• *Weighted visibility graph (WVG):* is a version of natural VG where edges between nodes are assigned weights based on the degree of visibility between corresponding data points (Supriya et al., 2016a).
• *Weighted horizontal visibility graph (WHVG):* is an advanced version of HVG that also considers the weight of edges and weakens the effect of nodes with long distances (Zhu et al., 2014b).
• *Power of scale-freeness visibility graph (PSVG):* states that the degree distribution of its nodes satisfies power-law, and hence, the extracted network has the scale-free property (Lacasa et al., 2008, 2009; Ahmadlou et al., 2012).
• *Multilayer visibility graph (MVG):* transforms multidimensional time series into multilayer networks for extracting high-dimensional information after analyzing the feature structure of the network (Nicosia et al., 2014).
• *Difference visibility graph (DVG):* where the edge and degrees are equal to the difference of edges and degrees of two VGs and HVGs with fixed nodes, which is very useful in acquiring the fundamental features of the signal (Zhu et al., 2014a).

The VG derived from brain timeseries data should be interpreted cautiously, as it does not necessarily reflect the true intricacies of the real brain’s network structure. This graph represents a simplified mathematical construct based on observable time series data, but it lacks a direct correlation with the actual connectivity between neurons or regions of interest within the brain. While the VG may reveal certain patterns or relationships within the time series data, it cannot provide insights into the specific neural connections, synapses, or the underlying physical architecture of the brain. It is a representation of data in a graph format, where edges and nodes are constructed based on mathematical algorithms, but these connections may not hold biological significance.

### 2.2. Visibility graph analysis

The visibility graph analysis is a straightforward and efficient method for time series data analysis that examines graph features. This method has numerous applications in brain analysis, providing valuable information on the fundamental characteristics of brain networks. By utilizing advanced network detection, prediction, and analysis techniques, this approach assists researchers in the field of brain diseases in gaining comprehensive and informative insights. As a result, this analysis method plays a crucial role in enhancing our understanding of brain network dynamics and in developing effective treatments for brain-related conditions. Through analyzing the local topological properties of the resulted networks in graph-theoretical area, we can extract valuable information about brain features.

Network analysis metrics can be defined based on different network features, including connectivity, centrality, and distance (Artameeyanant et al., 2017). Connectivity-based metrics include average degree, average clustering coefficient, modularity, and density, while centrality-based metrics measure dominance and closeness at the central point. Distance-based metrics, on the other hand, assess average shortest path length, global efficiency, and network diameter. The following sections examine the considered metrics. Other network analysis metrics, such as sparsity, density, small-world or scale-free properties, can be used to determine more precise network features.

Table 1 lists the most common metrics based on our article review. For instance, Ji et al. (2016) found that the degree distribution of the extracted brain network of individuals with job stress increases with the k coefficient, while that of normal individuals decreases with the *k* coefficient, or Wang et al. (2018), by analyzing brain network features, showed that the connections in the brain network of individuals with Alzheimer’s disease are more scattered than those of healthy individuals, indicating a scale-free property, and the range of connections in their brain network is reduced. Therefore, calculating and studying network analysis metrics in graph-theoretical area can be highly beneficial.

**TABLE 1:** Simple description of network analysis metrics.

Metric	Technical explanation
Degree	The number of connections a node has to other nodes.
Degree distribution	The probability distribution of degrees in the network.
Weighted degree average	The average weight of the edges in a node, calculated as the number of times an edge passes between a pair of nodes.
Average degree	The average number of edges per node in the graph.
Average shortest path length	The average number of steps along the shortest paths for all possible pairs of nodes in the network.
Average path length	The average path length, which is the average distance between a given node and all other nodes in the network, taking into account the number of times a node appears in the shortest paths between nodes.
Clustering coefficient	Measures the density of connections among a node’s neighbors.
Average clustering coefficient	The average number of common neighbors between a node and its neighbors.
Graph complexity index	A measure of overall complexity in weighted networks.
Global efficiency	The efficiency between two nodes, defined as the inverse of the shortest path length between them.
Local efficiency	The local efficiency of a node in the subgraph induced by its neighbors.
Density	The ratio of the number of edges in a graph to the maximum number of edges it could have.
Small world	A property for networks in which a path of the shortest possible length can be found between any two nodes.
Scale-free	In a scale-free network, small parts of the network are representative of the entire network, with a large number of nodes having low degree and a small number of nodes having high degree.
Modularity	The property of a network where connections are denser internally and sparser externally in relation to the enclosing module.

Visibility graph analysis has demonstrated utility in various applications within the field of brain research. Specifically, it can be categorized into three distinct areas of application as described below:

1. *Enhanced understanding:* The application of novel graph-based analysis techniques on time series data has opened up new avenues for comprehending complex brain dynamics. By investigating network properties such as centrality measures or clustering coefficients, researchers can uncover previously unknown concepts in the time series, such as crucial time points or regions that hold significance. We will refer to this property in the paper as VG analysis.
2. *Disease diagnosis:* VG analysis proves valuable in the classification of different states of disorders, such as Epilepsy, where two distinct states are present. Leveraging this analysis technique, it becomes possible to accurately classify and differentiate between various time series associated with different disorders, aiding in the diagnosis process.
3. *Disease prediction:* Utilizing time series data from individuals with specific disorders, VG analysis offers the potential to predict future changes or anticipate trends. By applying this analysis methodology, researchers can derive valuable insights into the trajectory of the disease, enabling early detection and intervention strategies.

## 3. Methodology

In this article, scoping review were conducted following the PRISMA methodology (Tricco et al., 2018). The eligibility criteria for selecting studies and the methods for collecting them were established and will be discussed in the upcoming sections.

### 3.1. Eligibility criteria

For the purposes of this scoping review, we established strict eligibility criteria to identify relevant studies for inclusion. Our search strategy targeted original research articles and conference proceedings on the topic of VG analysis. To ensure consistency and accuracy, we limited our search to studies published in English. We also sought to include studies examining the application of VG analysis in the context of brain disorders, including but not limited to Alzheimer’s disease, epilepsy, and fatigue. To ensure the quality and relevance of studies considered for inclusion, we excluded works in progress, editorials, dissertation papers, book chapters, and position papers. The application of these eligibility criteria allowed us to comprehensively identify and evaluate studies that met our research objectives.

### 3.2. Information sources and search strategy

To identify relevant studies for a scoping review, a comprehensive search was conducted between 4 January 2022 and 4 June 2022. Various platforms, including Google Scholar, Scopus, and search article functions provided by leading journal publishers such as Elsevier, Springer, and Taylor & Francis, were utilized to retrieve high-quality scholarly content from scientific journals, books, and conference proceedings. An advanced search technique with an “AND” condition was used to design the search strategy, focusing on the most relevant studies published between 2008 and 2023. Google Scholar search results were limited to the first 20 pages, and citing articles of the retrieved search results were also examined for potentially valuable articles. Date and topic filters were applied within Google Scholar to ensure efficient and precise search results. Scopus, the largest abstract and citation database, was instrumental in retrieving relevant studies. The initial search terms were “visibility graph analysis,” “EEG,” or “brain,” with advanced search filters limiting results to articles published from 2008 onward. The inclusion of article titles, keywords, and abstracts narrowed the search to 61 relevant articles, which were further refined to 53 articles and two doctoral dissertations based on full-text analysis.

The search article functions provided by eight leading journal publishers were also utilized to broaden the search for relevant articles and confirm the findings obtained from Scopus. This comprehensive search strategy, combined with careful selection criteria and filters, resulted in a thorough exploration of the literature, ensuring that relevant studies were included in the scoping review. It is worth mentioning that the year 2008 marks the inception of the VG concept by Lacasa et al. (2008), signifying the foundational period for the development of this scoping review research.

### 3.3. Study selection

**FIGURE 2:**
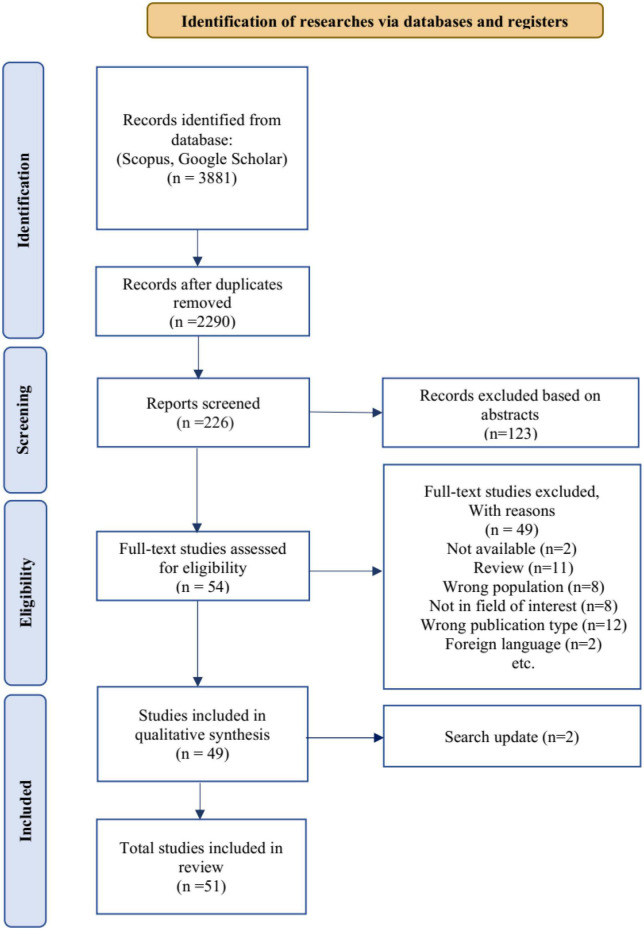
A PRISMA flow diagram illustrating the process of selecting relevant articles from reputable scientific databases. The variable “*n*” represents the number of articles at each stage.

Figure 2 illustrates the PRISMA diagram, which outlines the detailed process followed in this scoping review. All articles related to VG analysis in various brain disorders were identified from the selected databases. Subsequently, the abstracts of these articles were carefully reviewed to assess their suitability based on the predefined inclusion criteria. To ensure reliability and consistency, two researchers independently screened the abstracts. In cases where there were differences in opinion, the researchers engaged in thorough discussions and deliberations until a consensus was reached. During the abstract screening phase, the researchers scrutinized the content of each paper to determine its alignment with the inclusion criteria established for this study. This step was crucial in ensuring that only relevant articles were considered for further analysis. By involving multiple advisors and facilitating discussions, the review process aimed to minimize bias and enhance the reliability of article selection. The PRISMA diagram provides a visual representation of this systematic approach, demonstrating the systematic identification and screening of articles, as well as the collaborative decision-making process employed by the researchers. The diagram serves as a comprehensive overview of the study selection process, showcasing the rigorous and meticulous methodology followed in this scoping review.

## 4. Results and discussion

This section provides an overview of research findings on VG analysis in the brain, examined from various perspectives. To enhance the understanding and applicability of the results, we have utilized different visualization methods to present the findings in a concise and comprehensible manner within a compact format. Table 2 provides a summary of the section’s content, highlighting the comprehensive coverage of diverse aspects. The aim is to present the results in a manner that maximizes their utility and facilitates further investigation.

**TABLE 2:** Overview of the research results by summarizing the tables and figures captions and their visualization type.

Figures or table number	Different view of visibility graph analysis papers investigation	Visualization type
Figure 3	Year and country distribution of the papers	Line and stacked column chart
Figure 4	Journal and conference distribution of the papers determining the top publishers	Donut chart
Figure 5	General categorization of visibility graph analysis applications and associated disorders	Chord chart
Table 3	Association of visibility graph type with investigated brain disorder and related references	
Figure 6	Ratio of number of brain disorders investigated using visibility graph analysis	Waffle chart
Figure 7	Time distribution of visibility graph analysis application and associated disorders	Line and stacked column chart
Figure 8	Visibility graph types usages for each brain disorder	Stacked bar chart
Figure 9	Frequency of network metrics using in brain visibility graph analysis	Tree map
Figure 10	Popular evaluation metrics utilized in visibility graph analysis for brain disorders	Bowtie chart
Figure 11	Most commonly used machine learning algorithms by relative percentage	Brick chart
Figure 12	Commonly used keywords in brain visibility graph analysis	Horizontal bar chart

**FIGURE 3:**
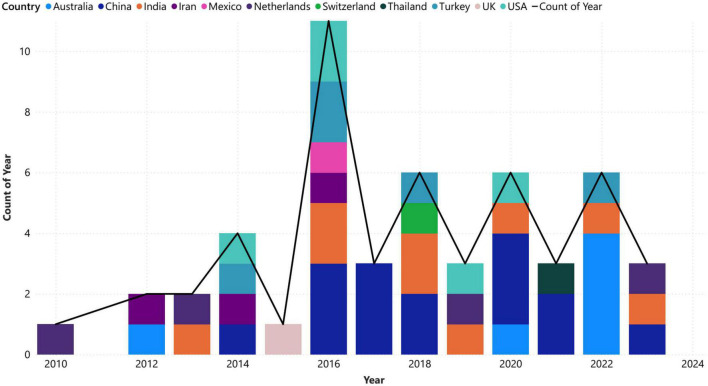
Number of published articles by year and country.

Figure 3 provides a timeline of analyzed articles by year and country, with the first article related to this field published in 2010. The chart shows that more than 85% of the published articles are from 2016 onward, indicating a growing interest in using VG analysis for brain networks in the future. China accounts for the majority of researchers in this field, contributing to approximately 10.14% of the article publications and emerging as the leading country in this field followed by Australia, India, and United States among others. The data also highlights the significant impact of the distribution of records among these countries on the overall distribution of records, as the countries with higher percentages have a greater influence on the distribution.

Also, the statistics in Figure 3 underscore the growing importance of VG analysis in the field of neuroscience and emphasize the need for continued investment in research and development to further explore this powerful analytical technique. The overall increasing slope of the growth line regarding VG analysis in brain networks indicates that this field is gaining momentum and will become even more important in the future. The trend in years, indicating sustained interest and activity in this area. Additionally, the emergence of the United States as a significant contributor to the field of graph analysis in recent years highlights the importance of continued investment in research and development in this area for developed countries.

The leading universities with a high number of publications, exceeding three papers, include Tianjin University from China, Izmir University from Turkey, and the University of Southern Queensland from Australia. Although the individual publication counts for each university may not be substantial, it is important to highlight the diverse representation of countries involved. Another diversity hints suggests a promising future for the proliferation of research and the emergence of interdisciplinary collaborations among researchers in various fields such as economy, medicine, and engineering regard to department and university research filed. Besides, Jiang Wang, Aydin Akan, and Yan Li have made the greatest contributions to publishing articles related to the analysis of brain VG. This suggests that these authors are prominent in this field and that their work has had a significant impact on the direction of research within the area of brain analysis. A statistical analysis of publication data of these authors may provide additional insights into the popular research directions. Consequently, researchers can use tools like bibliometric analysis to evaluate the productivity and impact of different authors, institutions, and fields of study, which can facilitate better decision-making in planning research strategies and collaborations.

**FIGURE 4:**
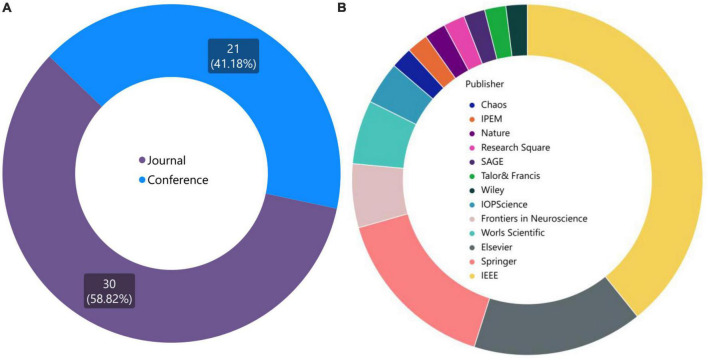
**(A)** Diversity of published articles in two categories: journal and conference. **(B)** Top publishers and journals in the field of brain graph analysis.

Prior to proceeding, it is intriguing to ascertain the distribution of publications across different journals specifically pertaining to VG research within the realm of neuroscience. Basically, the data presented in Figure 4 provide valuable insights into the distribution of publications in the field of brain graph analysis, highlighting the importance of different publication types and the impact of different publishers in this area. The pie chart in Figure 4A depicts the percentage of publications in journals and conferences. The fact that 21 conference papers were published from 51 published articles, suggests that conference proceedings are an important venue for disseminating research in this field. In addition to identifying the most popular journals for publishing research in brain graph analysis, the data also provides insights into the overall distribution of publications across different publication types. The dominance of IEEE, Springer, and ELSEVIER publications in this area highlights the importance of these publishers in the field of brain graph analysis, Figure 4B. This may be due to a variety of factors, such as the quality of the peer-review process, the reputation of the journals, or the accessibility of the publications. As more research is conducted in this field, it will be interesting to see how the distribution of publications evolves and how different publishers and publication types continue to contribute to the growth and development of this area. With a deeper investigation, it reveals that IEEE Xplore in conferences, and Physica A: Statistical Mechanics and its Applications in journals are the top publishers in this area.

Besides, a statistical investigation of the publication trends and patterns pertaining to VG analysis in brain networks, based on the number of authors and citations related to publishing articles reveals an average author count of 3.69 per article, indicating a relatively low level of collaboration among researchers in this multidisciplinary field. However, it is noteworthy that the maximum number of citations on a single paper is 323, which demonstrates the high level of interest and relevance of the topic. The impact of VG analysis in neuroscience research is exemplified by the study conducted by Zhu et al. (2014a), which utilized this approach to analyze sleep states. The resulting article has been cited in other studies a remarkable 323 times, underscoring the potential influence and significance of this method in the field. Moreover, the potential of VG analysis in collecting the researchers in a single team is demonstrated by another study focused on Alzheimer’s disease identification using the WVG approach and fuzzy learning (Yu et al., 2020). This study involved the participation of seven authors, highlighting the collaborative nature of research efforts in this area. In summary, the statistical data the citation and co-authorship data emphasizes the need for continued investment in research and development to further explore the potential of this powerful analytical technique.

**FIGURE 5:**
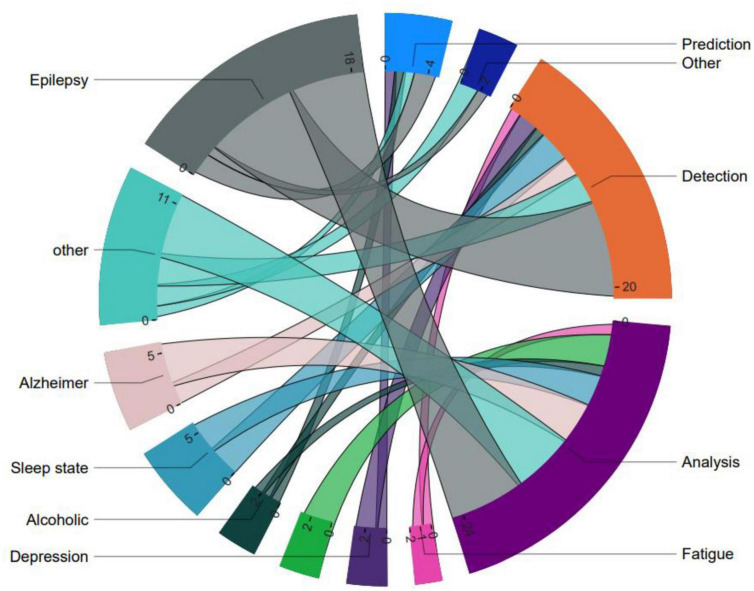
General categorization of visibility graph analysis applications and associated brain disorders.

Since VG analysis has been widely used in various fields for different purposes of processing time series data of brain activity, by classifying the application types, it was found that this approach is mostly used in three distinct fields of analysis, followed by diagnosis, prediction, plus other differentiated applications. Furthermore, the top seven categories of brain diseases studied using VG analysis were identified as Epilepsy, Sleep state, Alzheimer’s disease, Depression, Autism, Alcoholism, Fatigue, Down syndrome, and other conditions (Sengupta et al., 2013; Pei et al., 2014; Ahmadi and Pechenizkiy, 2016; Ji et al., 2016; Zhu et al., 2018; Samanta et al., 2019; Ozel et al., 2020; Cui et al., 2021; Varley and Sporns, 2021; Huang-Jing et al., 2023). Accordingly, Figure 5 illustrates the classification of these applications. It is evident that most of the research efforts have been directed toward the diagnosis of epilepsy, with relatively less attention paid to predicting other related areas of brain disorders. Furthermore, VG analysis has been most commonly applied in the context of Alzheimer’s disease following Epilepsy. These findings demonstrate the potential of VG analysis in contributing to the diagnosis and treatment of various brain-related diseases. Moreover, the results suggest that further research efforts are needed to explore the full potential of this analytical technique in other areas of brain diseases, such as depression and fatigue.

If we want to know more about the VG types that have been used to study each of the common brain disorders, we can create a table that lists each type of VG in a row, along with the brain disorders that have been studied using that type of graph and the related references. This would be similar to Table 3, but it would also include less common VG types that have only been used for special applications under category named other, such as WHVG-TE^1^ (Isfahani et al., 2022), WLPVG^2^ (Pei et al., 2014), 2DHVG^3^ (Huang-Jing et al., 2023), etc.

**TABLE 3:** Association of visibility graph type with investigated brain disorder and related references.

VG methods	Alzheimer	Epilepsy	Depression	ASD	Other	Sleep state	Fatigue	Alcoholic
VG	Ahmadlou et al., 2010; Cai et al., 2016; Wang et al., 2016b,^2018^	Zhu et al., 2012; Bhaduri and Ghosh, 2015; Hao et al., 2016a; Olamat et al., 2017, 2018, 2022; Wang et al., 2017, 2020; Xu et al., 2018; Mathur and Chakka, 2020; Tiwari and Wong, 2022; Villa Padilla et al., 2023	Bashiri and Mokhtarpour, 2022; Zhang B. et al., 2022	Wadhera and Kakkar, 2020, 2021	Pei et al., 2014; Ahmadi and Pechenizkiy, 2016; Hao et al., 2016b; Ji et al., 2016; Ozel et al., 2020; Varley and Sporns, 2021; Teymourlouei et al., 2023	Liu et al., 2016; Supriya et al., 2016, 2018; Xiong et al., 2019; Zhang X. et al., 2022	Gao et al., 2019	
HVG	Wang et al., 2018	Gao et al., 2016; Schindler et al., 2016; Wang et al., 2017; Xu et al., 2018; Isfahani et al., 2022; Tiwari and Wong, 2022; Villa Padilla et al., 2023	Bashiri and Mokhtarpour, 2022		Ahmadi and Pechenizkiy, 2016; Varley and Sporns, 2021; Huang-Jing et al., 2023; Teymourlouei et al., 2023	Liu et al., 2016; Supriya et al., 2016	Gao et al., 2019	Zhu et al., 2014a; Wang et al., 2016c
VGS		Wang et al., 2017; Olamat et al., 2018, 2022			Ahmadlou et al., 2013; Ozel et al., 2020		Sengupta et al., 2014	
LPVG	Cai et al., 2016; Wang et al., 2016b,^2018^	Gao et al., 2016; Xu et al., 2018			Pei et al., 2014; Hao et al., 2016b		Gao et al., 2019	
WVG	Cai et al., 2018; Yu et al., 2020	Wang et al., 2016a; Ebenezer Rajadurai and Valliyammai, 2018; Mathur and Chakka, 2020; Modak et al., 2020			Samanta et al., 2019	Supriya et al., 2018	Sengupta et al., 2014	Paranjape et al., 2023
PSVG	Ahmadlou et al., 2010	Bhaduri and Ghosh, 2015			Pei et al., 2014			
WHVG		Isfahani et al., 2022						
DVG		Wang et al., 2017	Bashiri and Mokhtarpour, 2022			Supriya et al., 2016	Varley and Sporns, 2021	
MVG				Wadhera and Kakkar, 2020	Samanta et al., 2019	Zhang X. et al., 2022	Gao et al., 2019	
Other		Isfahani et al., 2022			Pei et al., 2014; Ozel et al., 2020; Cui et al., 2021; Huang-Jing et al., 2023		Sengupta et al., 2014; Gao et al., 2019; Varley and Sporns, 2021	Zhu et al., 2014a

**FIGURE 6:**
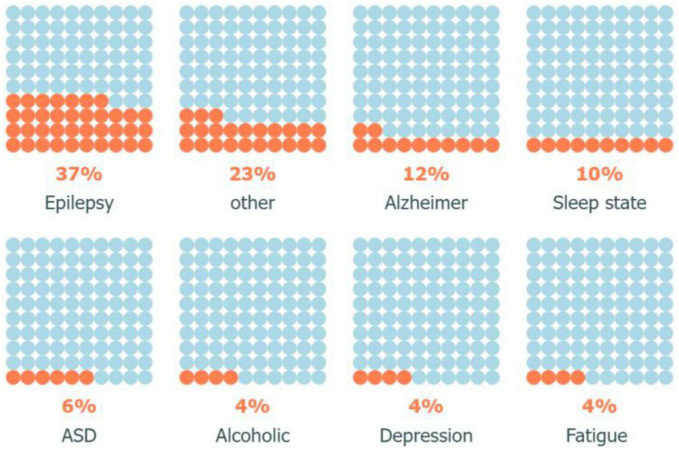
Ratio of disorders examined using visibility graph analysis.

By examining the VG analysis applications closely, we can clearly see that Epilepsy, and Alzheimer’s collectively account for approximately half of all investigated disorders, Figure 6. Epilepsy is a complex medical condition that has been the subject of extensive analysis and diagnosis by experts since 2010. It is evident from statistical data that Epilepsy continues to affect a significant number of individuals worldwide, leading to high morbidity rates and an overall decrease in quality of life. Besides, according to the Alzheimer’s Association, Alzheimer’s disease is the most common cause of dementia and accounts for 60–80% of dementia cases (Alzheimer’s Association, 2023). As such, researchers have been actively seeking ways to improve diagnosis and treatment options for these neurological disorders.

Moreover, the study of sleep disorders and autism has gained momentum in the research community due to their potential impact on cognitive function and overall health. With advancements in technology and increased awareness, experts hope to gain a better understanding of these conditions and develop effective interventions to improve patient outcomes (Distefano et al., 2023). Overall, the field of neurology continues to evolve rapidly, with new discoveries and innovations paving the way for improved healthcare delivery and patient care.

**FIGURE 7:**
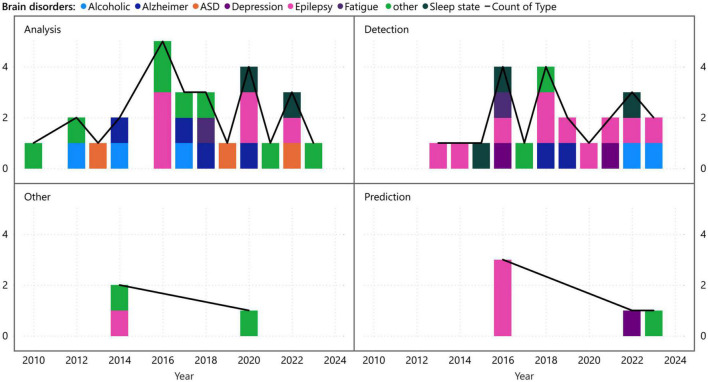
Time distribution of the different applications of visibility graph analysis associated with brain disorders.

A more detailed perspective stemming from Figure 5 involves the examination of brain disorders from various viewpoints, such as analysis, detection, prediction, and other aspects. Figure 7, which adopts this visualization approach, demonstrates that while analysis and detection have been the subject of ongoing research across different diseases over the years, prediction and other differentiated research on VG analysis, have received comparatively less attention. This disparity in focus could be attributed to the nature of brain time series data, where prediction may be considered less feasible than analysis and diagnosis. Alternatively, it could signify the need for further exploration and investigation of the potentials in the realm of prediction and other related areas.

**FIGURE 8:**
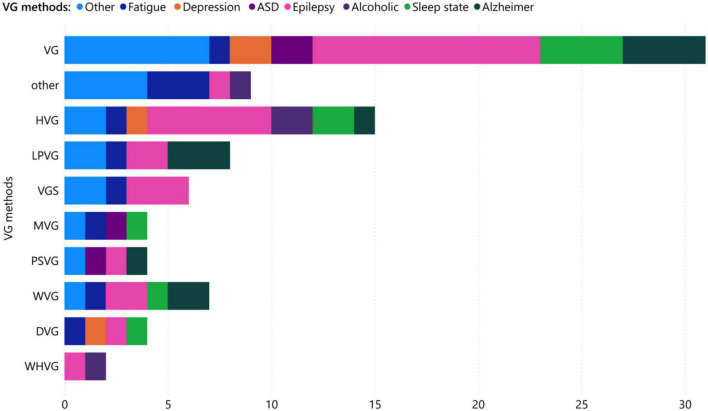
Different visibility graph shares related to each brain disorder.

Interestingly, VG analysis for brain is divided into several categories depending on the type of network used. Through our analysis of 53 research papers, we have identified the top eight most commonly used categories for VGs mentioned in Table 1 along with their applications, shown in Figure 8. Natural VG has attracted the most interest among researchers due to its simple and direct expression, and it accounts for the largest share of practical applications, approximately 36.36% of all papers. In addition, HVG has been widely used due to its substructure of simple graphs. VGS, followed by LPVG and WVG, have been the most popular types of VGs in the field of brain applications. While the epilepsy has been investigated mainly with VG and HVG, it is evident that most researchers in the field of Alzheimer’s disease have used VG, PSVG, LPVG, WVG, and HVG, which are among the top eight types of graph visualization. Additionally, researchers have focused on the use of VG, PSVG, and MVG in analyzing graph theory in the field of autism spectrum disorder.

**FIGURE 9:**
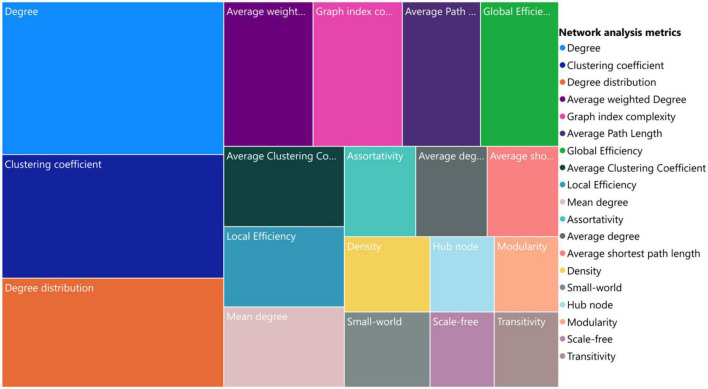
Commonly used network analysis metrics for brain visibility graph analysis.

During our review, we found that among the metrics used for network analysis, those listed in Table 2 were the most commonly used. Figure 9 demonstrates the tree map view of the metrics based on their usage frequency. It seems that for brain VG analysis, degree, clustering coefficient, and degree distribution are the three top leveraged metrics because they provide important information about the nature of the network in an easy-to-understand manner. These metrics can provide valuable insights into the structure and behavior of a network and are essential tools for understanding complex systems. These metrics have also been widely studied and applied in various fields. For example, degree centrality has been used to study social networks, communication networks, and biological networks, while clustering coefficient has been used to analyze brain networks and power grids. Degree distribution has also been used in the study of technological networks, transportation networks, and financial networks. Overall, the use of these metrics is critical for gaining a deeper understanding of complex systems and has become an essential part of network analysis.

**FIGURE 10:**
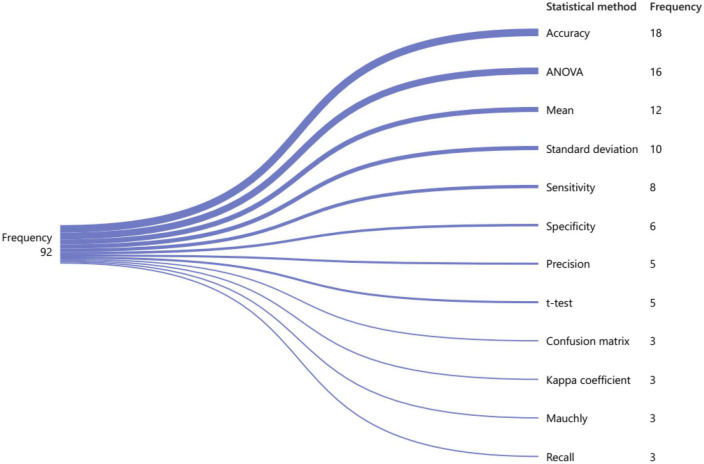
Commonly used evaluation measures for brain visibility graph analysis based on their frequency.

Researchers generally use statistical metrics to evaluate models. These statistical metrics are essential for evaluating the performance and effectiveness of models in network analysis. For example, accuracy is often used to measure the degree of agreement between predicted and actual values, while ANOVA is used to analyze variance between groups. Other statistical metrics such as correlation coefficients, regression analysis, and hypothesis testing can also provide valuable insights into the relationships between variables and the overall structure of the network. Accordingly, Figure 10 below shows 13 of the most commonly used statistical approaches in network analysis. Over 22 articles have used accuracy as a metric, while ANOVA has been used as a statistical metric in 19 articles. Additionally, 12 articles have employed statistical mean to evaluate their approach and model in their research. Some researchers have used more than one statistical measure simultaneously in one paper. These statistical techniques provide a powerful toolset for understanding networks and can help us gain new insights into the underlying mechanisms driving complex systems. Overall, statistical metrics are an indispensable component of network analysis and play a vital role in advancing our understanding of complex systems.

There are several popular machine learning classification algorithms that have gained significant attention and success in various domains such as brain. One such algorithm is the support vector machine (SVM), which aims to find an optimal hyperplane that separates different classes by maximizing the margin between them. SVMs are known for their ability to handle high-dimensional data and can effectively handle both linear and non-linear classification tasks through the use of kernel functions. Another widely used algorithm is Random Forest, which combines multiple decision trees to create a robust and accurate classifier. By aggregating the predictions individual trees, Random Forest can handle complex datasets, handle missing values, and provide feature importance rankings. Additionally, Logistic Regression is a simple yet powerful algorithm commonly used for binary classification tasks. It models the relationship between input features and the probability of belonging to a particular class using a logistic function. Logistic Regression is computationally efficient, interpretable, and can handle large datasets. Moreover, *K*-nearest neighbors (KNN) is a non-parametric machine learning algorithm that classifies new data points based on their proximity to the labeled examples in the training set. These are just a few examples of the popular machine learning classification algorithms that have proven their effectiveness in solving a wide range of classification problems (Dash et al., 2021).

**FIGURE 11:**
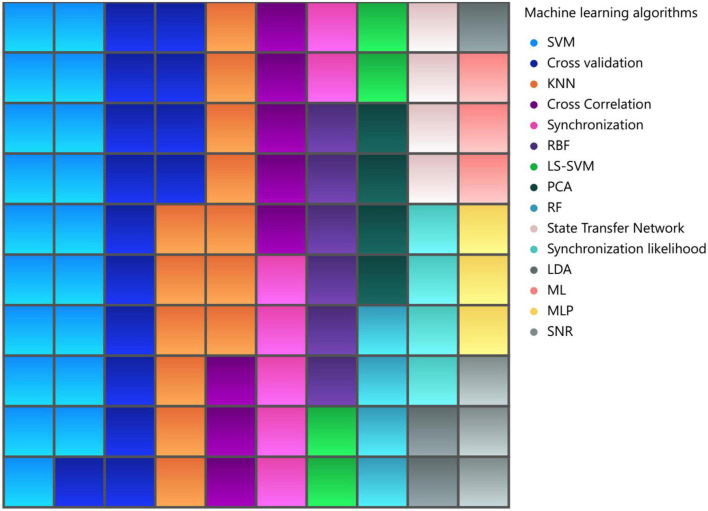
Most commonly utilized machine learning algorithms in visibility graph analysis.

During our literature review, we collected information on the machine learning methods used in brain visibility network analysis, Figure 11. In 22.22% of the articles, SVM was used as a machine learning approach for classification tasks, while KNN was used in 12.5% of the articles, and RBF (radial basis function) was used in 6.94% of the articles. It is interesting to note that dimensionality reduction techniques such as LDA (linear discriminant analysis) and PCA (principal component analysis) were also used in some of the articles. These methods are crucial for reducing the complexity of high-dimensional data and extracting meaningful features from it. Overall, the use of machine learning and dimensionality reduction techniques in brain VG analysis has provided new avenues for exploring complex systems and has significantly influenced the field’s research direction, especially for diagnosis applications of the brain disorders.

**FIGURE 12:**
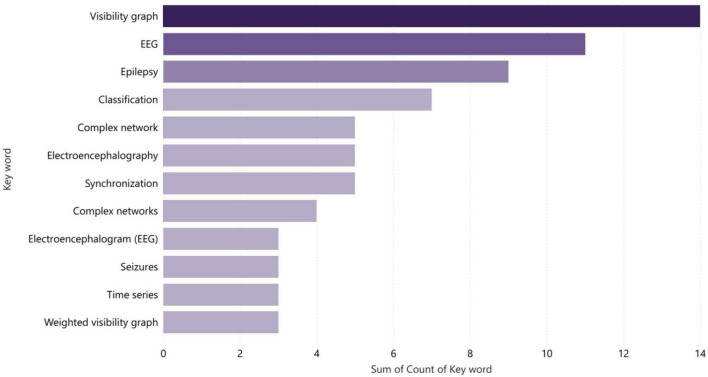
Most commonly used keywords in reviewed articles.

During the process of reviewing articles, the most frequently used key terms were “Visibility Graph,” “EEG,” “Complex Networks,” and “Epilepsy” in that order, as indicated in Figure 12. This suggests that these concepts are currently dominant and prominent in research related to the field being studied. Statistical analysis of the frequency of these terms can provide insights into the trends and patterns of research in this area, and help researchers identify important topics and areas for further exploration. Additionally, understanding the relationships between these key terms can provide valuable information about the underlying structures and mechanisms of the phenomena being studied. Additionally, researchers can use this information to identify important areas for further exploration and research. This type of statistical analysis serves as a useful tool for researchers seeking to stay up-to-date with the latest trends and developments in their field.

Clearly, the utilized data in the research on VG analysis of brain diseases can provide valuable insights and clues. Also, the sample size is a crucial factor in any study or analysis, as it can affect the accuracy and reliability of the results. Therefore, researchers must carefully consider their sample sizes to ensure the validity of their findings. Our investigations show the highest number of data samples in brain VG analysis were used for sleep-related studies (Xiong et al., 2019), 295, while the lowest number of data samples were used for epilepsy studies (Modak et al., 2020), 5. Likewise, the youngest participant in the sampling process was 1.5 years old, which was related to the epilepsy dataset. On the other hand, the oldest participant in the sampling process was 85 years old, which was related to the Alzheimer’s dataset. It is also interesting to note that the maximum number of samples among women was 28, which was used in an article (Yu et al., 2020) focusing on Alzheimer’s disease detection.

In comparison, brain VG is more popular with EEG timeseries data than fMRI data. This is because EEG data is a continuous signal that is recorded over time, while fMRI data is a discrete signal that is collected at a series of time points. The continuous nature of EEG data makes it more suitable for the construction of VGs, as it allows us to capture the temporal dynamics of brain activity.

Furthermore, EEG data is less expensive and more portable than fMRI data, making it more accessible to researchers. This has led to a wider use of EEG data in studies of brain connectivity, including the use of VGs. However, VGs can also be used with fMRI data. In fact, there are some studies that have shown that VGs can be used to identify functional connectivity in fMRI data (Sannino et al., 2017). However, these studies are still in their early stages, and more research is needed to determine the effectiveness of VGs for fMRI data.

## 5. Conclusion and future works

Writing a scoping review for brain VG analysis is of paramount importance for several reasons. Firstly, it provides a comprehensive overview of the existing literature, identifying the breadth and depth of the research conducted in this field. This helps to identify gaps in the current knowledge and areas that require further investigation. Secondly, it aids in clarifying key concepts and theories used in brain VG analysis, thereby enhancing understanding and facilitating communication among researchers. Thirdly, it helps to identify the methodologies and tools used in previous studies, which can guide future research design. Lastly, a scoping review can help to identify the potential impacts and applications of brain VG analysis in various fields such as neuroscience, psychology, and clinical medicine, thereby informing policy and practice.

This article presents a comprehensive review of VG analysis in order to determine the practical and research diversity in the field of brain analysis. The study examined 51 articles and identified a significant number of publications in this area. On average, four articles were published each year over the course of 13 years. VG analysis can improve the diagnosis and prediction of brain diseases. The most common use of this method has been for diagnosing epilepsy. However, other brain diseases, particularly Parkinson’s disease and attention deficit hyperactivity disorder (ADHD), have received less or no attention in graph analysis research. Future studies may identify the reasons for these gaps in research and address them or starting new studies on not investigated brain disorders.

The article acknowledges two limitations in its findings. First, it is possible that relevant published articles may have been excluded due to our search terms not being reflected. The second limitation is due to the lack of use of other databases such as PubMed, ScienceDirect, Semantic Scholar, Web of Science, and IEEE Xplore.

Future work in this field can focus on the integration of deep learning and graph machine learning techniques into brain VG analysis. The complexity and non-linear nature of brain networks necessitate the use of advanced machine learning methods that can capture these characteristics. Deep learning, with its ability to learn hierarchical representations, can be used to extract meaningful features from brain VGs. These features can then be used to classify different brain states or to predict outcomes in neurological disorders. On the other hand, graph machine learning, which is designed to work with graph data, can be used as an advanced technique of processing the VG with extra value-added information. The combination of these two approaches can lead to a more comprehensive understanding of brain signals and their role in health and disease. This will require the development of new algorithms and computational tools, as well as the collection and analysis of large-scale brain network data. The results of this research could have significant implications for the diagnosis and treatment of neurological disorders. Eventually, the image visibility graph (IVG), which is a novel approach for transforming images into VGs in a distinctive way (Iacovacci and Lacasa, 2020), could potentially find application in the realm of neuroscience, particularly given the abundance of neuroimaging data accessible. This technique might offer a fresh perspective for analyzing brain images, enabling the construction of a network representation that captures the interconnections and communication patterns present within these images of the brain. Furthermore, IVG analysis may help identify disruptions or abnormalities in these images, aiding in the diagnosis and understanding of neurological disorders. In summary, the utilization of Image VGs in brain research has the potential to revolutionize our comprehension of brain function and dysfunction, offering new avenues for both basic neuroscience research and clinical applications.

## Author contributions

ZS: Data curation, Writing—original draft, Visualization. SS: Conceptualization, Validation, Formal analysis, Writing—review and editing, Supervision.

## Footnotes

1 Weighted horizontal visibility graph-transferable entropy.

2 Wavelet limited penetrable visibility graph.

3 Two-dimensional horizontal visibility graph.
